# COVID-19 Tests the Market Stability Reserve

**DOI:** 10.1007/s10640-020-00441-0

**Published:** 2020-08-04

**Authors:** Reyer Gerlagh, Roweno J. R. K. Heijmans, Knut Einar Rosendahl

**Affiliations:** 1 Department of Economics, Tilburg University, Tilburg, The Netherlands; 2 School of Economics and Business, Norwegian University of Life Sciences, Ås, Norway; 3 Oslo Centre for Research on Environmental friendly Energy (CREE), Oslo, Norway

**Keywords:** COVID-19, EU ETS, MSR, Environmental policy, H23, Q41, Q54, Q58

## Abstract

We compare the decrease in energy demand and CO2 emissions in Europe during the financial crisis 2008–2009 with the expected drop in demand and emissions due to COVID-19, and the price response of the EU Emission Trading System (EU ETS). We ask whether the rather limited current price reduction may be due to the Market Stability Reserve (MSR), implemented in the EU ETS between the two crises. Stylized facts and basic theory are complemented with simulations based on a model of the EU ETS. Together, they suggest a mixed result. The MSR stabilizes the EU ETS price in turbulent times, but imperfectly. We show that the more persistent the COVID-19 shock is, the less the MSR is able to serve its purpose.

## Introduction

The COVID-19 pandemic brought global economic activity to a sudden halt in the first half of 2020. The result was a substantial fall in energy demand. By the end of April 2020, the IEA (2020) expects global energy demand to fall by 6 percent in 2020, and global CO2 emissions to drop 8 percent. In the Europe Union, impacts are expected to be even greater, with energy consumption falling by 11 percent. As about half of EU’s CO2 emissions are regulated by the EU Emission Trading System (EU ETS), demand for emission allowances (EUAs) will likely fall along with lower emissions, and so, one expects, will EUA prices. Indeed, when the 2008 financial crisis hit, the EUA price dropped by more than 50 percent in only a few months. This observation contrasts with the 2020 COVID-19 crisis, since at the time of writing the EUA price has fallen less in 2020 even though the IEA expects the impact on EU emissions to be “almost double the impact of the global financial crisis”.

The present paper discusses whether a recent regulatory change in the EU ETS can explain the seemingly different price response in 2020 vis-à-vis 2008. In 2015, the EU introduced the Market Stability Reserve (MSR), the mechanics of which were crucially revised in 2018. The purpose of the MSR is to “address the current surplus of allowances” and “improve the system’s resilience to major shocks by adjusting the supply of allowances to be auctioned”.^1^ The COVID-19 pandemic is indeed a “major shock” (see Sect. 2), and the question is whether the MSR is living up to its expectations.

So, how does the MSR work? The precise nature of the MSR is explained in Perino (2018) and Gerlagh and Heijmans (2019), and we wil summarize it in Sect. 3. Essentially, the MSR decreases the (cumulative) supply of EUAs when a sustained shortage of demand for EUAs occurs, stabilizing prices in the process. In Perino’s (2018) words, this temporarily and partially punctures the waterbed,^2^ and a negative shock in demand can reduce cumulative emissions over time.^3^

Section 4 develops a simulation model to quantitatively connect our theory to empirical observations. By means of our model we investigate the effects of a negative shock in demand, comparing outcomes with and without the MSR. We identify a pertinent issue: while the MSR succeeds in absorbing small shocks almost perfectly, large and persistent shocks—the ones most in need of absorption – are not dealt with by the MSR mechanisms. Section 5 discusses these findings and connects them to calls for a green recovery strategy.

Our paper fits into a broader literature on the effect of wider economic phenomena on emissions and prices within EU ETS. In its early years of operation (Phase I), EUA prices were driven by macroeconomic developments (Chevallier 2011) and fuel prices (Hintermann 2010). Koch et al. (2014) find that economic activity was a robust explanator of EUA price dynamics from 2008 to 2013. Moreover, even as the wider economy recovered after its plunge in 2008, EUA prices remained low (Ellerman et al. 2016). Indeed, the financial crisis has been found to be a structural break in the EUA price time series, characterized by a sharp drop with long-lasting effect (Zhu et al. 2015). We report on the consequences of COVID-19, the next big economic crisis.

## Comparing Demand Shocks

**Fig. 1:**
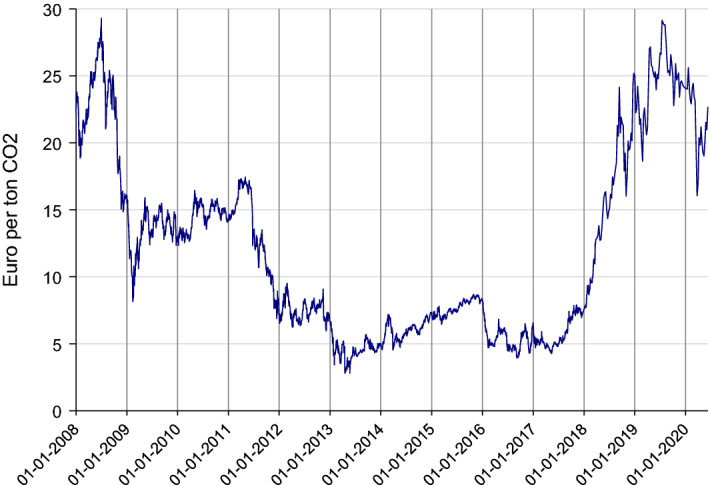
EUA (EU ETS) price 2008–2020

When the financial crisis hit the global economy in the middle of 2008, the EUA price responded quickly. It decreased from a high of 25 Euro per ton at the end of August to a low of less than 10 Euro in February 2009, before stabilizing around 13 Euro for the next year, see Fig. 1. The EUA price likely decreased as market participants anticipated lower economic activity and hence reduced demand for EUAs (emissions) in EU ETS. When the financial crisis was followed by a recession in many EU countries, the EUA price fell further and stayed below 10 Euro throughout the years 2012–17.

When the COVID-19 pandemic hit the world in the beginning of 2020, and most countries in the world closed down large parts of their economic activity, the EUA price also fell, but not nearly as much as in 2009 (Fig. 1). From January/February to May 2020, the price decreased by some 20 percent (from around 24 to 19 Euro per ton), but returned to pre-Corona levels by early June 2020.

The weaker price response in 2020 might be explained by a lower negative shock in emissions. But is it? Are market participants expecting less of an impact now than they did over a decade ago? Or are there other reasons why the price has fallen less this time? It is not easy to answer these questions as global COVID-19 responses are still unfolding, yet it will be useful to consider some relevant indicators and forecasts.

The most natural indicator to start with are emissions themselves. Emissions regulated by the EU ETS declined by 3 percent from 2007 to 2008, and by an additional 8 percent in 2009 (EEA 2019). The degree to which these reductions were driven by the financial crisis rather than emission regulation itself is hard to assess. After 2009, emissions continued to decline along with the EU recession, but much more gradually. Since EUA prices decreased as well, the observed decrease in demand is probably a lower bound for the structural decrease in the demand function.^4^

There is little doubt that the COVID-19 pandemic will reduce EU ETS emissions in 2020, but at the time of writing it is hard to know how much. According to the IEA (2020), CO2 emissions in the EU fell by 8 percent in the first quarter of  2020 (vis-a-vis Q1 2019). Bt the end of April, IEA (2020) expects a reduction of the EU’s energy consumption of 11 percent in 2020 vis-à-vis 2019, and “almost double the impact of the global financial crisis”. A forecast for CO2 emissions in the EU is, unfortunately, not provided.

Considering EU ETS emissions specifically, Refinitiv (2020) expects these emissions to drop by 14 percent in 2020, which is 17 percent compared to their previous (pre-Corona) forecast for 2020. They also expect significantly lower emissions after 2020 than previously expected, in total 1.3 Gt in the years 2020-2030 (close to 10 percent on average over this decade). IEA (2020) does not provide forecast beyond 2020, but write: “Even in 2021, global economic activity might well be below the 2019 level”. Taken together, data and forecasts from the IEA and Refinitiv suggest that the negative shock in emissions due to COVID-19, at least in the short run, will be bigger than in 2008-09. The longer term is more uncertain. For this we have to consult other indicators.

Indicators that are documented as good proxies for changes in demand for ETS allowances include GDP and fossil fuel prices, see Hintermann (2010), Chevallier (2011), Creti et al. (2012), Koch et al. (2014), Zhu et al. (2015), and Ellerman et al. (2016). For comparison of the two crises, we focus on crude oil because it is traded in large volumes. Its price responds quickly to changes in the (forecast of) economic activity. In 2008, it fell from all-time high of 140 USD per barrel (Brent blend) in the summer of 2008 to around 45 in the beginning of 2009. It recovered quite quickly though, and the average price in 2010 was slightly above the average price in 2007 (around 75 USD per barrel). In the first months of 2020, the Brent blend price has been halved.^5^ While the oil price reflects current demand relative to current capacity, the expectations of future oil prices are aggregated through stock prices for major oil companies, measuring the expected persistence of the crisis. Shares of Royal Dutch Shell and BP fell in both crises by somewhat less than 50 percent.^6^ Exxon Mobil share prices did not decrease as much as the others in 2008, but fell from around 70 USD at the end of 2019 to around 45 USD in May 2020. However, by the end of May and early June 2020, all three oil companies saw their share prices rise again.

Summing up, when evaluated in March 2020, evidence collected suggested a decrease in demand for EUAs during and after the COVID-19 crisis that is at least of the same order and persistence as that in 2008, while end of May 2020 long-term expectations became somewhat more optimistic. Yet the EUA price in EU ETS dropped less in March 2020 than during the 2008 crisis, and almost fully recovered end of May. There might be various reasons, as the EU ETS today is not the same as it was ten years ago. Expectations about the future of the ETS are different. We will focus on a natural candidate for the different price responses: the Market Stability Reserve (MSR), introduced as part of the EU ETS between the financial crisis and the COVID-19 pandemic. In the next section, we explain how the MSR works and how it smoothens the price response in a demand crisis.

## The Market Stability Reserve

In 2015, the EU established a Market Stability Reserve (MSR) as part of the EU ETS, which in 2018 was substantially revised. Each year in which banked EUAs exceed 833 $\hbox {MtCO}_2$, the number of auctioned EUAs next year is reduced.^7^ EUAs that are not auctioned are instead moved into the MSR. The number of EUAs that enter the MSR equals 24% of banked EUAs (12% as of 2024, according to EU regulation). When banking drops below 400 $\hbox {MtCO}_2$, EUAs corresponding to 100 $\hbox {MtCO}_2$ are taken out of the MSR, and added to the auctioned volumes next year.

The major revision of 2018 lets EU ETS cancel allowances in the MSR. This happens when the number of EUAs in the MSR exceeds the number of auctioned EUAs of the previous year, in which case all EUAs in excess of this threshold are permanently canceled. Canceling EUAs in the MSR, which then cannot return to the market, effectively reduces the cumulative cap on emissions in the EU ETS; cumulative supply of EUAs has become endogenous. For more details about the MSR, see Perino (2018) and Gerlagh and Heijmans (2019).

**Fig. 2:**
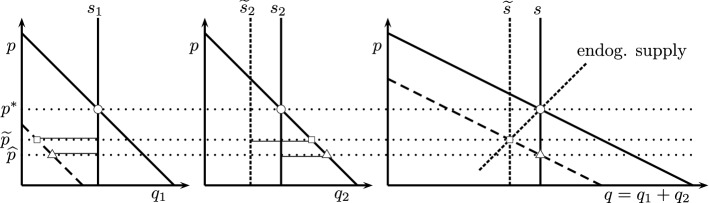
Equilibrium selection .Circles as equilibrium marker prior to demand shock, triangle for post-demand shock without MSR, square for post-demand shock with MSR. Dashed downwards sloping lines indicate demand function after shock. Vertical dashed lines denote endogenously adjusted supply. Lines on top of price lines denote banking, lines below denote the emptying of the allowances bank. Dashed upward-sloping line in right panel denotes implicit supply curve for first-period demand shocks that do *not* spillover to second period

The workings of the MSR can be conceptually illustrated in a simple two-period framework, see Fig. 2. The two leftmost panels show the equilibrium in the first and second period, while the rightmost panel displays the intertemporally integrated equilibrium. Initially, for expositional convenience, we assume equal supply and demand functions in the two periods, given by the vertical and downwards sloping solid lines, respectively.

Now assume that a negative demand shock hits the market in period 1. If banking is allowed, we obtain a new equilibrium with lower prices in both periods (${\widehat{p}}$). Demand is reduced in period 1 and the excess allowances are used in period 2, with aggregate demand unchanged. This is the waterbed effect: If emissions are lower at one point in time (or space), they go up at another point in time (or space). In Fig. 2, the triangle indicates the adjusted equilibrium. The waterbed dampens price volatility: a 10 percent reduction in demand over one or a few years, either 2009 or 2020, becomes a much smaller relative cumulative demand reduction when put in perspective of the full time horizon of the EU ETS. We therefore should not expect large price swings based on contemporary demand shocks. Reversing the logic, one can interpret the observed large price swings as strong evidence for a very small price elasticity of demand for allowances.

Next, add the MSR. As explained above, increased initial banking reduces future supply of allowances and thus aggregate supply, indicated by the negative shift in supply (${\widetilde{s}}$) in period 2 (Fig. 2). In the right panel, we also illustrate the supply effect by drawing an upwards sloping endogenous supply curve. Importantly, the slope of the endogenous supply curve depends on the relative size of the demand shocks. If a demand shock is symmetric over both periods, so that prices drop but banking does not change, the MSR will not interfere and supply is unaffected. That is, the endogenous supply curve is vertical, as if there were no MSR.^8^ The more demand is reduced in period 1, the more allowances will be banked, and the bigger the shift in supply. As a consequence, the price is reduced, but less than without the MSR (${\widetilde{p}}>{\widehat{p}}$), meaning the effect is stronger than a mere waterbed effect. The relative effectiveness of the MSR is a matter of quantitative analysis, for which we resort to simulations in the following section.

## Model Simulation

To simulate the ETS market with and without the MSR, we use a stylized, dynamic, and deterministic model, see Gerlagh et al. (2020). The model incorporates key details of the MSR. In our baseline scenario (before the COVID-19 shock), the price begins at 21.0 Euro per ton CO2 in 2019 and increases with the interest rate of 5 percent. Exogenous supply of EUAs declines linearly to zero in 2057, whereas endogenous demand (i.e., emissions) drops to zero in 2067.^9^

We next construct a version of our model without the MSR but with lower exogenous supply of EUAs, so that the starting price in 2019 is the same as in the model version with the MSR. Since emissions only depend on the ETS price, this means the (expected) emission paths are the same in both versions of the model and before a shock is implemented.

The future effects of COVID-19 on demand for EUAs is uncertain, especially the long-term effects. For this reason, we construct three alternative scenarios that have the same short-run effects (2020–22), but different long-term effects, see Table 1 which shows annual reductions in demand at given ETS prices. In the “Short” scenario, there are no impacts on demand after 2022. The difference between “Long and medium” and “Long and big” is the size of demand reductions after 2030.^10^ The last column of the table shows cumulative reductions in demand, given the baseline price path. We notice that cumulative demand is almost five times higher in “Long and big” than in “Short”. As the model is deterministic, there is perfect foresight within each scenario. We discuss the implications of this assumption in the next section.

**Table 1:** Annual and cumulative reductions in demand for allowances

	**2020**	**2021**	**2022**	**2023–30** (annual)	**2031–66** (Annual)	**2020–66** (Cumulative)
**Short**	260	195	130	0	0	**585**
**Long and medium**	260	195	130	73	20	**1872**
**Long and big**	260	195	130	73	50	**2859**

*Note*: Demand changes at given baseline equilibrium price, in $\hbox {[MtCO}_2]$

**Fig. 3:**
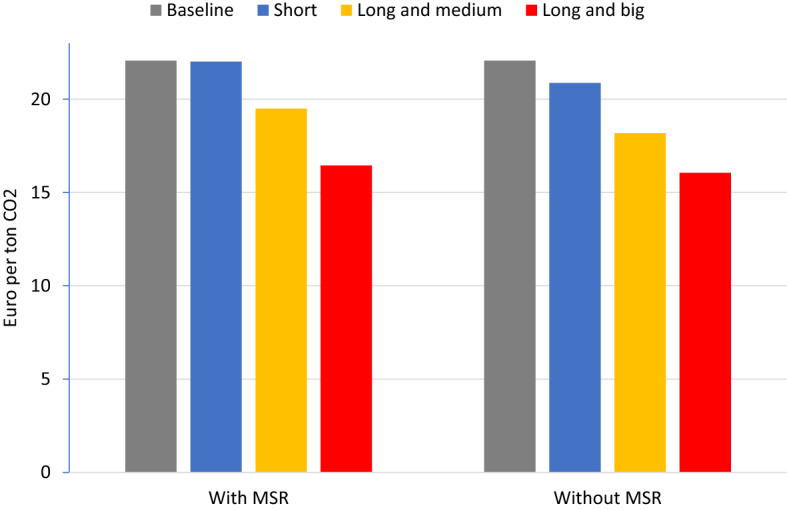
Price in 2020 in different scenarios with and without MSR

Before examining the effects of the MSR, it is constructive to consider how the demand reductions play out in the model without MSR. Since supply of EUAs is then fixed, the waterbed effect is fully operative and hence cumulative emissions are the same with or without the COVID-19 shock. To bring demand in line with constant supply, the ETS price has to come down; the price reduction, shown in Fig. 3, is approximately proportional to the reduction in cumulative demand shown in Table 1. In the “Short” scenario, the price drops by 1.2 Euro, while in the “Long and big” scenario it drops by 6.0 Euro. In all scenarios, emissions are higher after 2030 than in the baseline scenario, stimulated by lower ETS prices.

**Fig. 4:**
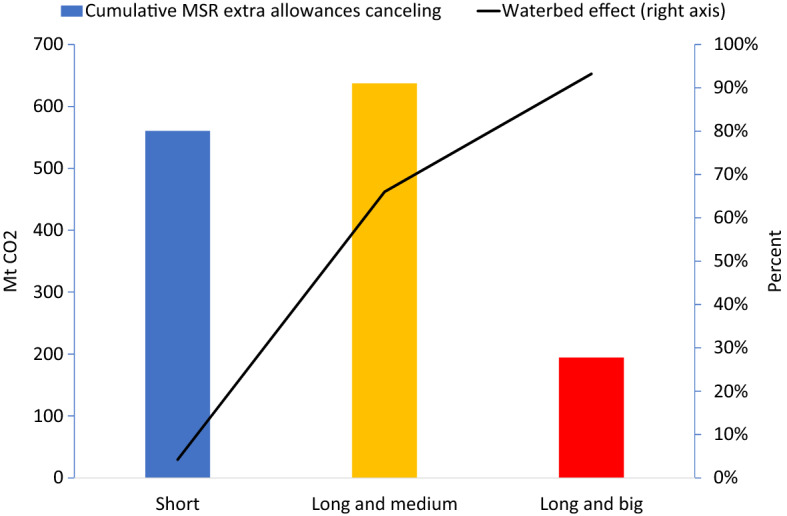
Extra canceling through MSR and waterbed effect

**Fig. 5:**
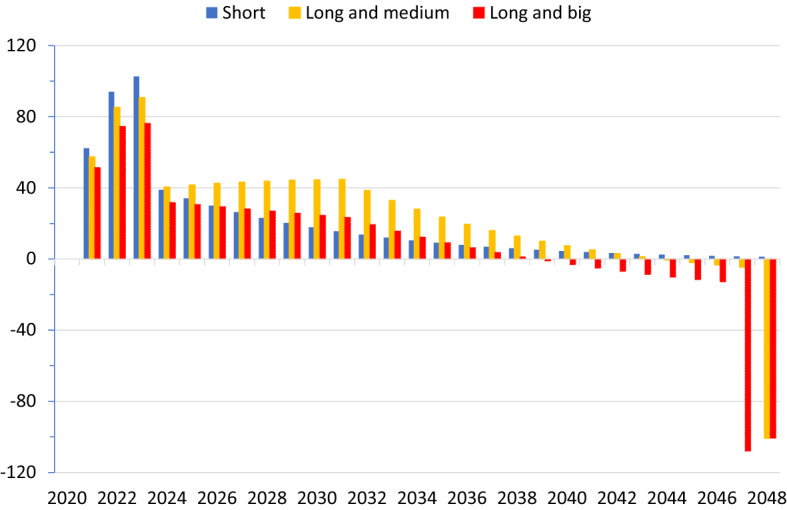
Inflow into the MSR. Differences vis-a-vis the baseline scenario

Next we consider the effects of the MSR. In the “Short” scenario, reduced demand for EUAs in 2020-22 leads to more banking of EUAs. By 2024, after three years of 24 percent intake, 56 percent of these additional banked EUAs have entered the MSR. In the later years, almost all of the additionally banked EUAs eventually enter into the MSR (see Fig. 5 in the Appendix) and get canceled. Cumulative supply of EUAs is reduced almost one-to-one with the drop in demand through the MSR; cumulative additional canceling amounts to 560 Mt (Fig. 4, first bar), only slightly below the direct drop shown in Table 1, implying that there is only a 4 percent waterbed effect (Fig. 4, right axis). This is why the ETS price hardly changes (Fig. 3, second vs. first bar).

In our “Long and medium” scenario, the reduction in demand is more persistent. Future demand reductions, however, have a lower propensity to flow into the MSR. The extra MSR canceling is less then 80 Mt (Fig. 4, second vs. first bar), while cumulative demand is decreased by almost 1300 Mt more than in the “Short” scenario (Table 1, second vs first row). The waterbed effect now amounts to 66 percent (Fig. 4, second y-axis). Taken together, the price in 2020 falls by 2.6 Euro, as compared to a drop of 3.9 Euro without the MSR (Fig. 3).

In the “Long and big” scenario, the impacts of future demand reductions are even stronger. The reduced future demand reduces banking, and thus the inflow into the MSR, which now works ‘in reverse’. Due to a lower intake, fewer EUAs get canceled, from 640 Mt in the “Long and medium” scenario to only 190 Mt in the “Long and big” scenario (Fig. 4, third vs second bar). The waterbed effect is back and almost fully operational at 96 percent (Fig. 4, second y-axis). The price in 2020 falls by 5.6 Euro, i.e., almost as much as the 6 Euro drop without the MSR (Fig. 3).

## Discussion and Conclusions

Observations suggest that the 2020 drop in demand induced by the COVID-19 pandemic exceeds that resulting from the 2008 financial crisis. Nonetheless, EAU prices fell less in 2020 than in 2009; by June 2020, allowance prices were almost back at the level of January. In this sense, the MSR works well: it stabilizes the market. However, the extent to which the market is stabilized is very sensitive to long-term expectations, even more so than before the MSR. To understand these differential effects, we investigate whether the permanence of a demand drop might be a factor governing the success of the MSR when it comes to stabilizing EU ETS. We observe that the MSR partly fails to do its job. Theory and our simulation model show a strong dampening price effect of the MSR on short-lived demand shocks; the MSR effectively cancels the perturbations of EUA demand. On the other hand, for long-lived demand shocks both our theory and simulations show the MSR to be less effective, or even ineffective. If the market anticipates future demand to decrease as much as present demand, there is a price drop without any reallocation of EUAs over time. The MSR is ineffective in such cases. If, however, the market is more optimistic and expects the crisis to be deep but temporary, the MSR does an almost perfect job stabilizing the market.

The simulation model has various limitations. The three main shortcomings are that we assume perfect foresight, no changes in context variables such as the rate of return on assets, and no difference between short- and long-run price responsiveness.^11^ We also assume no changes in (expected) policy measures that might affect EU ETS directly or indirectly.

Prices in EU ETS also depend on expectations about regulation. We can draw some policy lesson about that from our analysis. Consider policies that are complementary to the ETS and aim at a green recovery, such as an accelerated green transition. If these lead to stronger emissions reductions in the short run than in the long run, the MSR boosts the effectiveness of this policy by taking out additional allowances. On the other hand, if an accelerated green transition reduces future emissions more than current emissions, the MSR will partly reverse such policies as decreased banking leads to reduced cancellation; that is, the cumulative amount of available allowances goes up.^12^

Years of practice and experience with EU ETS seem to have led to a change in perspective. The EU ETS was constructed as a cap-and-trade system because certainty over quantities was perceived as more important than certainty over prices. Ample experience with a volatile allowance market has lead to the MSR, which while it does stabilize prices also opens the door to manipulation by unregulated parties.^13^ We think policy makers and market participants alike have become increasingly appreciative of stable prices. The next big improvement of EU ETS may well come through the introduction of price collars (Flachsland et al. 2020), or regulations that combine price and quantity information (Gerlagh and Heijmans 2018; Pizer and Prest 2020).

## Footnotes

1 https://ec.europa.eu/clima/policies/ets/reform_en

2 If the emissions cap is fixed and binding, any supplementary policies will not affect total emissions, but only shuffle emissions around (Böhringer and Rosendahl 2010). This is often referred to as the waterbed effect.

3 Besides the studies mentioned above, there are quite few published studies examining the effects of the revised version of the MSR so far. Bruninx et al. (2020) use a long-term investment model to examine the effects of the MSR. In a short paper, Bruninx and Ovaere (2020) simulates potential impacts of the COVID-19 pandemic. Compared to the current paper, they do not contrast with the 2008 crisis, and their analysis is rather brief. There are more studies of the first version of the MSR, see e.g. Kollenberg and Taschini (2019), Fell (2016) and Salant (2016).

4 Whereas the review of the EU ETS in Martin et al. (2016) suggests quite moderate effects on emissions in the second phase, Dechezleprêtre et al. (2018) finds 15 percent emission reduction.

5 Prices of coal and natural gas in Europe have shown a quite similar pattern as the crude oil price, both in 2008 and 2020. One difference between the two periods is that both prices were at very high levels when the crisis hit in 2008, while they were already at very low levels at the start of 2020. Still, the prices have fallen significantly, and in the second half of May 2020 the UK gas price fell below 10 p/th for the first time since 1999 (www.barchart.com/futures/quotes/NF*0/interactive-chart).

6 Royal Dutch Shell’s share dropped from around 80 USD at the end of 2007 to around 45 at the end of 2008, and from around 60 USD at the end of 2009 to 30–35 USD by May 2020. BP dropped from around 70 USD to around 40 USD during the credit crisis, and from around 40 USD to below 25 during the COVID-19 crisis.

7 To be precise, auctioned EUAs are reduced from September the following year to August the year after. The EU has introduced the term “Total number of allowances in circulation (TNAC)” (EU Commission 2019), which for our purpose is equivalent with private banking of EUAs.

8 Demand shifts in period 2 will in fact have the opposite effect on supply, resulting in a downwards sloping endogenous supply, see Gerlagh et al. (2020) and Rosendahl (2019).

9 This follows from our model calibration, see Gerlagh et al. (2020) for more details.

10 The reduction in 2020 is consistent with Refinitiv (2020)’s revised forecast of ETS emissions in 2020, while the cumulative reduction in 2020-30 in the two last scenarios is consistent with Refinitiv’s revised forecast for the whole decade. It is not clear to what degree Refinitiv takes into account any dampening effects from lower EUA prices in their forecast. If they do, it follows that the negative shock in demand in their analysis is higher.

11 As price responsiveness is likely higher in the long-run than in the short-run, our model may overestimate the initial price responsiveness and hence underestimate the canceling of EUAs in the persistent scenarios.

12 For more on this “green paradox”, see (Rosendahl 2019; Gerlagh et al. 2020).

13 See Gerlagh and Heijmans (2019) for a formal analysis; German NGO ForTomorrow puts the theory laid out in that paper to practice.
